# Hopping the Hurdle: Strategies to Enhance the Molecular Delivery to the Brain through the Blood–Brain Barrier

**DOI:** 10.3390/cells13100789

**Published:** 2024-05-07

**Authors:** Sinnead Anne Cogill, Jae-Hyeok Lee, Min-Tae Jeon, Do-Geun Kim, Yongmin Chang

**Affiliations:** 1 Dementia Research Group, Korea Brain Research Institute, Daegu 41062, Republic of Korea; sinneadcogill@dgist.ac.kr (S.A.C.); eprince1015@kaist.ac.kr (J.-H.L.); jmt1986@kbri.re.kr (M.-T.J.); 2 Department of Brain & Cognitive Sciences, Daegu Gyeongbuk Institute of Science and Technology (DGIST), Daegu 42988, Republic of Korea; 3 Department of Bio and Brain Engineering, Korea Advanced Institute of Science Technology (KAIST), Daejeon 34141, Republic of Korea; 4 Department of Molecular Medicine, School of Medicine, Kyungpook National University, Daegu 41944, Republic of Korea; 5 Department of Radiology, Kyungpook National University Hospital, Daegu 41944, Republic of Korea

**Keywords:** neurodegenerative disease, blood–brain barrier, drug delivery

## Abstract

Modern medicine has allowed for many advances in neurological and neurodegenerative disease (ND). However, the number of patients suffering from brain diseases is ever increasing and the treatment of brain diseases remains an issue, as drug efficacy is dramatically reduced due to the existence of the unique vascular structure, namely the blood–brain barrier (BBB). Several approaches to enhance drug delivery to the brain have been investigated but many have proven to be unsuccessful due to limited transport or damage induced in the BBB. Alternative approaches to enhance molecular delivery to the brain have been revealed in recent studies through the existence of molecular delivery pathways that regulate the passage of peripheral molecules. In this review, we present recent advancements of the basic research for these delivery pathways as well as examples of promising ventures to overcome the molecular hurdles that will enhance therapeutic interventions in the brain and potentially save the lives of millions of patients.

## 1. Introduction

The brain is the most crucial organ involved in controlling the physiological and cognitive functions of the body. Of importance, these physiological functions of the brain are sophisticated and well maintained through the electrophysiological buffering system that preserves neuronal function. This system is maintained by the unique vascular structure, namely the blood–brain barrier (BBB). The BBB selectively allows the entry of molecules from the peripheral system and protects the brain from any insult from the peripheral system such as infection, as well as maintaining the microenvironment of the brain [1]. Leakages of vascular factors, such as circulating immune cells and coagulation factors, into the brain were observed in the brains with neurological disorders indicating the loss of barrier roles of BBB [2,3]. The entry of circulating factors into the brain parenchyma causes neuroinflammation and neurodegeneration [1]. Therefore, damage to the BBB is most often related to the development of neurological disorders such as multiple sclerosis (MS) or neurodegenerative diseases (ND) such as Alzheimer’s disease (AD) and Parkinson’s Disease (PD) [4,5,6]. Thus, the importance of maintaining the BBB integrity is emphasized to preserve the disease progress. The barrier role of BBB is a hurdle for the delivery of therapeutics against neurological diseases into the central nervous system (CNS). Only molecules that are selected by brain endothelial cells can pass through BBB. The fate of drug delivery was destined by the transport system on brain endothelial cells. For example, transferrin receptor delivers transferrin from the blood to the brain through receptor-mediated transcytosis (RMT) [7]. However, efflux pumps, such as multidrug resistance protein 1 (MDR1), expressed in brain endothelial cells are able to return the drug to the blood [8]. So, the delivery of therapeutic agents to the brain presents another hurdle due to the existence of transporters and/or junctional proteins that are highly expressed on the brain endothelial cells that line the BBB, dramatically reducing the delivery of pharmaceutical reagents to the brain. Hence, effectively increasing the delivery of therapeutic reagents to the brain is one of the most important themes in drug development for neurological and neurodegenerative diseases [9,10].

Multiple approaches have been implemented to increase the permeability of the drugs, including manipulating the BBB’s integrity through utilizing chemical insults, modulating signaling pathways and the modifications of drugs [11]. Some of these approaches have proven to be successful but the majority demonstrated limited success in increasing drug delivery while some even show severe side effects including irreversible damage to the BBB integrity. Issues stem from a lack of understanding of the underlying mechanisms of how the BBB increases permeability to the peripheral molecules and how one may manipulate these pathways to enhance the molecular delivery to the brain. In this review, we aim to present the structure and basic physiology of the BBB, existing approaches to enhancing BBB permeability, as well as the recent advances in BBB research that can be applied to increase drug delivery to the central nervous system (CNS). Finally, we are going to suggest future recommendations that might enable the efficient delivery of molecules to the brain.

## 2. Structure and Function of the BBB

**Figure 1:**
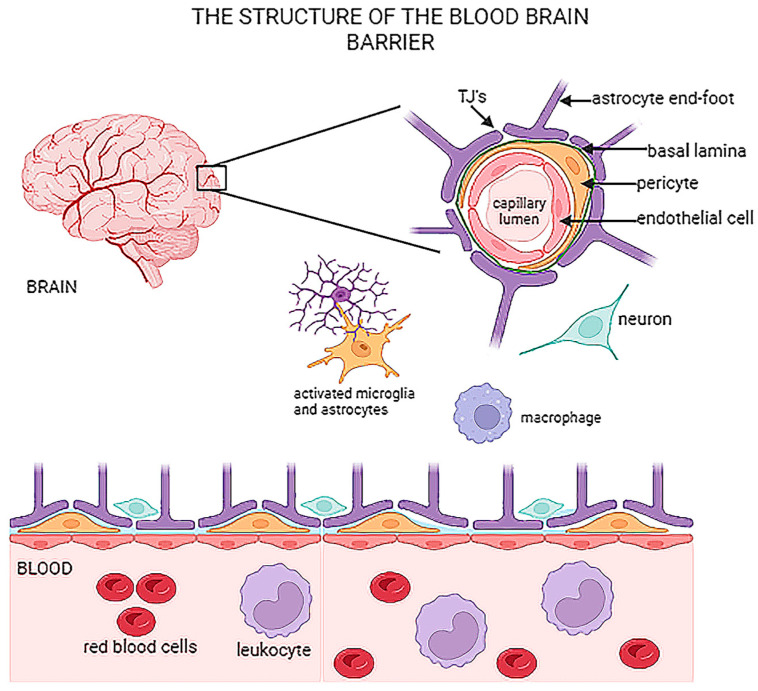
Schematic representation of the basic structure of the BBB, cross-sectionally and cell structure including astrocytes, pericytes, endothelial cells, TJs and associated neurons. TJs—tight junctions.

To reiterate the most important function of the BBB, the system blocks the entry of unwanted molecules into the brain, thus acting as a gate keeper for the maintenance of the normal physiology of the brain. To achieve this, the BBB has a unique structure. First, brain endothelial cells construct the vascular wall to divide the blood luminal side and brain parenchyma. The gaps between brain endothelial cells are sealed with tight and adherens junction proteins; these include claudins, occludin and accessory proteins such as Zonula occludens (ZO) [12,13]. These junctional proteins synergistically increase the physical resistance of the BBB to block the movement of large molecules across the BBB [13]. Moreover, cellular components, including pericytes and astrocytes, are enclosed in the vascular wall and can notably enhance the resistance of the BBB [14,15,16] so that it reaches mega angstrom levels. Figure 1 below demonstrates the basic structure of the BBB.

Structural changes in the BBB are related to autoimmune diseases such as MS and microvascular injuries such as strokes [17]. Junctional loss in the BBB is a marker of BBB disruption [18], which leads to microbleeding. Pericyte loss was observed in brains with neurological diseases including MS showing BBB disruption [19]. Pericyte loss is related to BBB damage. Pericyte depletion causes a loss of BBB function, resulting in an increase in permeability blood factor [20]. In addition, astrocytes that construct the BBB also changed in brains with MS. The experimental autoimmune encephalomyelitis (EAE) animal model showed a reduction in astrocyte endfeet in the brain [21].

Brain endothelial cells select the molecules to transport across the BBB. The selection is regulated by diverse receptors, channels, efflux pumps and transporters expressed on brain endothelial cells. These transport systems are divided as described below.

### 2.1. The Paracellular Pathway

Traditionally, it has been believed that the molecular passage through the cell-to-cell junction is not viable due to the existence of the tight junction proteins. Indeed, it is well known that the BBB does not allow the entry of small molecules that are polar or molecules that are larger than 500 Da [22,23]. Some molecules that have smaller molecular weights can cross the barrier through tight junctions, but even in such cases, many small molecules are substrates for the drug transporters. To regulate this process, the existence of tight junction proteins blocks the entry of molecules through the paracellular pathway [24,25,26]. The process is more prominent in the case of pathological conditions, where molecules are delivered through the paracellular pathway where the BBB can provide protection against toxic molecules and peripheral factors such as fibrinogen, immunoglobulin, and complements that are known to be immunogenic in the brain [1]. Additionally, in the cases of severe pathological conditions, the entry of peripheral molecules that can elicit immune responses has been confirmed through several studies. For example, in cases of multiple sclerosis, the damage to the paracellular pathway that is mediated by the activation of immune responses induces the entry of peripheral immune cells, including CD-4 T cells and macrophages, into the brain [27,28]. The consistent entry of peripheral molecules, such as fibrinogen, exacerbates the damage to the tight junctions [29]. These processes are mediated by the activation of matrix metalloproteinases (MMP), which degrade the junctional molecules, and secretion of cytokines that result in junction disruption by the activation of Rho-GTPase [30,31]. It is also well known that the activation of Rho-GTPase through cytokine release can disrupt the barrier integrity via the activation of Rho-GTPase, thereby enhancing the cell-to-cell junction damage [32]. Once the cell-to-cell junctions are disrupted, several mechanisms are implemented to alleviate the damage through the reversal of junctional gaps by inducing the formation of cortical actin (e.g., sphingomyelin-induced recovery of the cell-to-cell junction).

### 2.2. The Transcellular Pathway

Brain endothelial cells can inhibit the transport of peripheral molecules into the brain. It is commonly believed that transport is mediated through the blockade of the entry of molecules by the formation of cell-to-cell junctions. However, recent studies have demonstrated that these pathways are mediated through the activation of the endo-lysosomal degradation pathway that selectively recognizes the molecules that can cross the BBB and molecules that should be degraded before their entry into the brain [33,34,35]. Conventionally, brain endothelial cells allow the entry of molecules through RMT or through fluid-phase-dependent molecular pathways [36,37,38]. For RMT, molecules are internalized via clathrin-mediated endocytosis (CME) that encompasses 95% of cells [39,40]. Brain endothelial cells consist of abundant coated pits that are structurally characteristic for the CME, where examples of RMT include transferrin receptors and insulin receptors [41,42]. For the fluid-phase transport, it has been reported that the caveolae have important roles in the delivery of molecules across the barrier, where one of the regulators for this system is known as major-facilitator-superfamily-domain-containing protein 2 (MFSD2A) [33,43]. An experimental example of this factor is the MFSD2A mice that showed suppression of caveolae pathways and, as a result, reduced uptake of dextran in the brain, indicating a crucial role of the fluid-phase transporting system for the non-receptor-mediated transporting system [44]. It is still unclear whether caveolae can independently mediate transcytosis or if they simply deliver molecules to endosomes to mediate further delivery pathways that require further study.

Molecules that enter the early endosome pathway have been shown to diverge into the early endosomal trafficking pathway or the late endosome–lysosome degradation pathway, where non-essential molecules are readily degraded [45]. This is a crucial mechanism for the RMT system. One of the main uncertainties in this context, however, is how internalized molecules can be sorted and determined to be transported into the brain. For example, even with a similar molecular structure, antibodies binding to the transferrin receptor showed varying capabilities to cross the BBB [46], an indication that specific molecular structure is a key determinant for the fate of a molecule. Subsequently, this fate occurring through the endosomal trafficking pathway delivery is determined by sorting tubules, demonstrating that the interaction with proteins in the cytoplasm may determine the capability of a molecule to go through transcytosis [34]. Sorting tubules consist of multiple proteins including sorting nexins with the BAR domain (Bin/Amphiphysin/Rvs domain) and additional proteins, including Arf6GAP [47,48,49]. However, the mechanistic understanding of how this machinery can differentiate the molecules that can cross the barrier is still elusive. The utilization of sorting tubules in the molecular delivery process and sorting process has been reported in multiple molecules including cation-dependent mannose-6-phosphate receptor (CD-MPR) and single-domain llama antibodies (FC5) that form part of the camelid antibody construct [50,51]. Molecules that have passed the security check at this point of the process will then pass through the basolateral side of the brain endothelial cells, going through exocytosis steps. Despite existing knowledge, how this process is mediated and final secretions from the membrane are under investigation [52,53]. Moreover, the transcytosis mechanism for molecular delivery across the brain endothelial cells is yet to be elucidated. The foundational information that is still missing for these delivery systems would be particularly enlightening in the development of technologies and therapeutic delivery mechanisms to the brain that may be the starting point for future topics of study in the field of drug delivery to the CNS system.

### 2.3. Transporters of the BBB

**Figure 2:**
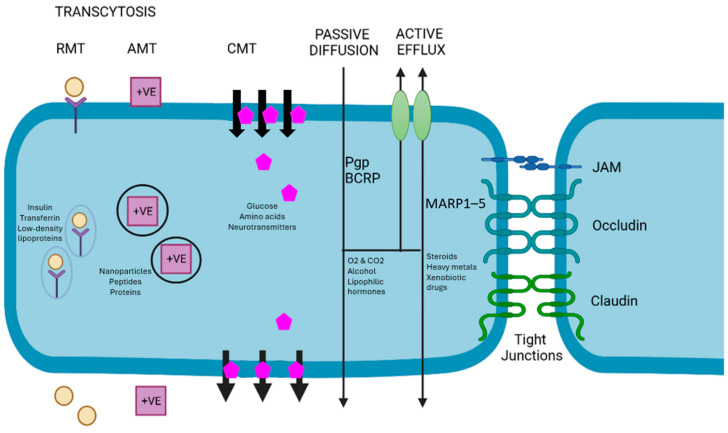
Schematic representation for various modalities of transport across the BBB. RMT—receptor-mediated transport; AMT—adsorption-mediated transport; CMT—carrier-mediated transport; JAM—junctional adhesion molecules; Pgp—P-glycoprotein; BCRP—breast cancer resistance protein; MARP1–5—muscle ankyrin-repeat proteins 1–5. Adapted from Pulgar et al. [23].

While a wide variety of lipid-soluble molecules can diffuse passively through the BBB, molecules such as xenobiotics and endogenous metabolites rely on active efflux to be transported across the BBB into the brain. This process involves the working of efflux pumps with specific transporters which actively move substrates into the brain. Figure 2 illustrates the various methods of BBB transport. Of importance are the efflux transporters, namely the adenosine triphosphate (ATP)-binding cassette (ABC) transporters and solute carrier (SLC) transporters of the BBB. This review will focus more on ABC transporters, which consist of a molecular pump on the cellular membrane with two transmembrane domains and two cytosolic domains, existing at the ATP binding site [54].

#### 2.3.1. Solute Carriers

SLC transporters, which include facilitative transporters and secondary active transporters, are responsible for the transport of both charged and uncharged molecules. Currently, there are over 400 members of the SLC transporter family and they are divided into 66 families [55,56], which regulate the transport of amino acids, neurotransmitters, inorganic ions, vitamins, organic anions, energy sources and lipids [57]. Large neutral amino acid transporter 1 (LAT1), also known as SLC7A5, interacts with SLC3A2 to form CD98. LAT1 is expressed in diverse tissues, including those in the BBB, and it facilitates the exchange of intracellular amino acids and large neutral amino acids across the cell membrane. A crucial member of the SLC2A family is known as the glucose transporter (GLUT). Glucose, an energy source as a substrate for glycolysis, is transported by the SLC transporters across the plasma membrane. GLUT1, also known as SLC2A1, regulates glucose uptake into the brain endothelial cells and is widely expressed in these cells as well as the brain capillaries. Throughout glucose metabolism, some internalized glucose is utilized for glycolysis and in the brain endothelial cells, while some leak into the brain parenchyma. Glucose effluxes are therefore controlled by GLUT density in the basolateral membrane and the gradient of glucose concentration between endothelial cells and brain parenchyma [58,59,60] and GLUT1 expression in brain capillary is evidently essential for brain homeostasis. Veys and colleagues reported that GLUT1 inhibition by endothelial cells showed impairment of glucose metabolism in vitro and endothelial cell-specific GLUT1 deficiency led to loss of angiogenic properties in the neonatal mouse brain in vivo [61]. In addition, endothelial cell-specific GLUT1 knock-out in adult mice showed neuronal loss and neuroinflammation [61]. Moreover, the expression of glucose transporter (GLUT1) and transferrin receptor (TrfR) expressed on the brain endothelial cells can allow entry of peripheral nutrients into the brain. In several studies, it has been demonstrated that the dysfunction of these transporters is related to the progression of brain diseases, including AD and PD [62,63].

#### 2.3.2. ABC Transporters

##### Basic Biology of ABC Transporters

**Figure 3:**
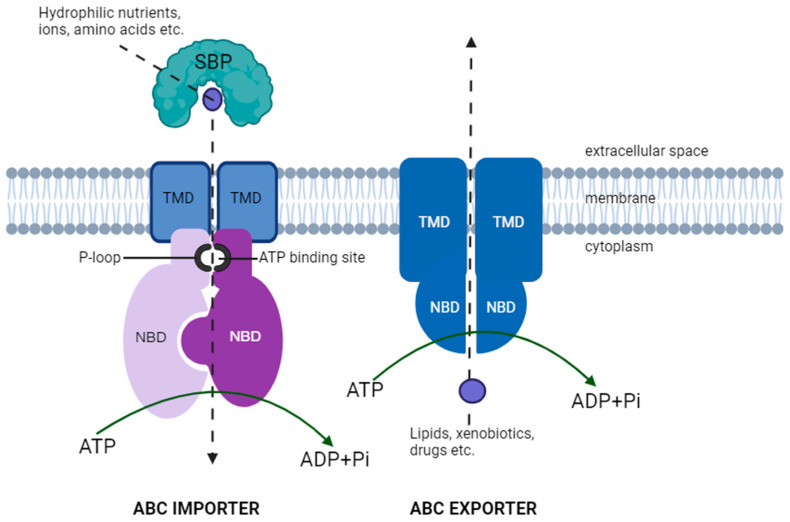
Basic structure of ABC transporters, whereby through ATP hydrolysis, causes a conformational change that drives the substrate across the membrane. TMD—transmembrane domain; NBD—nucleotide-binding domain; ATP—adenosine triphosphate; ADP—adenosine diphosphate; SBP—substrate-binding protein. Adapted from CUSABIO (https://www.cusabio.com/Transmembrane/A-transport-machine-ATP-binding-cassette.html (accessed on 30 December 2023)).

The ABC transporter family includes P-glycoprotein (P-gp), breast cancer resistance protein (BCRP) and organic anion transporting polypeptide (OATP). These proteins utilize the energy of ATP hydrolysis to translocate solutes across the BBB [64,65]. P-gp and BCRP, which are expressed on the luminal side of the brain endothelial cells, are responsible for the active pumping of compounds out of the endothelial cells back into the blood. P-gp, also known as multi-drug resistant protein 1 (MDR1) or ABC sub-family B member 1 (ABCB1), is a representative ABC transporter protein and is involved in cellular multidrug resistance by acting as an efflux pump to interrupt drug accumulation accompanying anti-cancer resistance [66]. P-gp is highly expressed in brain endothelial cells and acts as an efflux pump in the BBB to restrict the invasion of circulating factors [67]. One of the widely accepted views regarding its mode of action is its role as flippase utilizing ATP as its energy source before its entry into the cytoplasms of the brain endothelial cells [68]. To evaluate the role of P-gp in the efflux of drugs into the brain endothelial cells, drugs such as ivermectin, which is an anti-parasitic drug, have been shown to increase the expression of the MDR1 gene in the mouse brain model,, which is a transcript for P-gp [69]. Its activity has thus been targeted for a potential increase in medicinal effects and is considered a major hurdle for drug delivery into the brain [70]. Many chemotherapeutic agents (e.g., Gemcitabine and Doxorubicin) that are effective in in vitro culture systems for brain cancers are not effective in vivo, since these drugs are transporter substrates for P-gp [71,72]. Figure 3 illustrates the basic structure of ABC transporters.

##### Mechanisms to Increase Function and Expression Level of ABC Transporters

It is important to regulate the expression level of P-gp to enhance the delivery of drugs to the brain. P-gp is known to be regulated by various signaling pathways, e.g., (1) it is activated by the nuclear receptor expressed in the brain endothelial cells, (2) ligand binding to the transporter that consequently activates signaling pathway mainly mediated by nuclear factor kappa-light-chain-enhancer of activated B cell (NF-κB) nuclear translocation, and (3) modulation of basal activity of transporters.

For nuclear-receptor-mediated upregulation of P-gp, metabolites or drugs that can bind to the DNA binding motif induce the translocation of nuclear receptors to the nucleus and consequently induce the binding of receptors to its partners and form the heterodimer that can bind to DNA for transcription initiation [73]. It is widely accepted that activation of these receptors can be induced by various types of chemicals that are binding to the pregnane X receptor (PXR), constitutive androstane receptor (CAR), aryl hydrocarbon receptor (AhR), vitamin D receptor (VDR) and glucocorticoid receptor (GR). For receptor-signaling-mediated regulation of the modulation of P-gp expression, inflammation is one of the key drivers inducing increased expression of P-gp [74]. Receptor signaling of tumor necrosis factor-α (TNFα), a well-known cytokine, is one of the examples that can induce overexpression of P-gp in the brain endothelial cells [75]. Activation of TNFR1 induces the release of endothelin 1 (ET-1), thereby activating protein kinase C, which is responsible for the translocation of NF-κB, subsequently triggering enhanced expression levels of P-gp [76]. Oxidative stress is another regulator for the expression level of P-gp. Increased oxidative stress triggers activation of nuclear factor (erythroid derived 2)-like 2 (Nrf2) [77]. Furthermore, rats exposed to sulforaphane (SFN), which is an activator for oxidative stress, showed activation of Nrf2, and sulforaphane could cause an increased expression of P-gp in the brain endothelial cells [78].

##### Mechanisms Which Decrease Function and Expression Level of ABC

There are multiple pathways that reduce the function of the ABC transporters without affecting their expression level, and this is one of the major modulating methods to enhance the drug delivery to the brain for ABC transporter substrates. For example, at low doses, vascular endothelial growth factors (VEGF) decrease the activity of P-gp through its regulation of fetal liver kinase 1 (flk-1) and Src kinases both in vitro and in vivo and enhance the accumulation of P-gp substrates such as verapamil without causing damage to junctions of the brain endothelial cells [79]. At higher doses, VEGF can disrupt the junction of the BBB and proves to not be a useful tool to modulate the P-gp activity [80]. However, finding the downstream targets of VEGF that regulate the function of P-gp would be a valuable avenue for P-gp modulation. According to more recent studies, it has been shown that inhibition of the mammalian target of rapamycin (mTOR) can induce the downregulation of P-gp, transiently mediated by activation of the Akt-dependent pathway. Although this pathway has shown promise in downregulating the function P-gp, it has selectivity issues, and safety concerns that need further study to fine tune its applicability. This includes the application of Estradiol (E2), as it has been known to downmodulate the expression level of BCRP [81,82]. In following mechanistic studies, it was shown that Estradiol (E2) blocks association with the plasma membrane that would reduce its capacity to pump out drugs from cells at an early time point in treatment. In longer-term experiments (6 h post-treatment), the expression level of BCRP was been decreased, which is mediated by estrogen receptor β (ERβ) that signals by phosphoinositide 3-kinase (PI3-K), Akt [83]. Alternatively, E2 can induce the activation of Wnt/β-catenin pathways, which play a role in adult tissue homeostasis that further degrades the BCRP protein [84,85]. Overall, we could conclude several molecular pathways are closely associated with the regulation of the expression and function of these cell surface associated transporters in the brain endothelial cells.

##### Attenuation of Drug Efficiency by ABC Transporters

The efficiency at which drugs are delivered to the brain and the rest of the body is determined by pharmacokinetics and pharmacodynamics. Pharmacokinetics is described as the absorption, metabolism, and excretion of drugs in the body, and these processes are controlled by transporters expressed on the cellular membrane. For brain disorders, drug entry into the brain from the circulatory blood is the first hurdle in drug efficiency. As mentioned above, some ABC transporters (e.g., P-gp) act as efflux pumps to inhibit the accumulation of substrates in cells and are expressed in various cells including brain endothelial cells [86,87]. These transporters recognize many drug chemicals as potential transport substrates and inhibit their delivery into the brain [88]. For example, Irinotecan is an anti-cancer drug for the treatment of glioblastoma, a type of brain tumor observed in the adult brain. A difference was found in the efficiency of the delivery of Irinotecan into the brains of mice that lacked MDR1. The detection of Irinotecan was shown to be higher in the group that lacked MDR1 in the brain as compared to the littermate control [89]. In addition, drug delivery is also regulated by the activity of ABCG2, also known as BCRP. When co-treated with MBL-II 141, an inhibitor against ABCG2 [90], studies detected the enhanced delivery of Irinotecan into the brain [90]. Another example, namely Dasatinib, a common treatment for leukemia, shows its anti-cancer effects via the inhibition of Akt and ERK1/2 signaling pathways in neuroblastoma cell lines [91]. Dasatinib also acts as a substrate for MDR1, where MDR1-expressing cells exhibited the limitation of Dasatinib accumulation in cells [92]. Moreover, the detection of Dasatinib in the brain is very poor, as demonstrated within 3 h following a tail vein injection of Dasatinib. In MDR1a/b and BCRP1 triple-knockout mice, however, the group showed a much higher brain concentration of Dasatinib than the wild-type mice [92]. Milciclib, a CDK inhibitor with potential neoplastic activity, can affect brain tumor growth in vivo and in vitro [93]. Despite evidence of anti-tumor efficacy in the brains of wild-type mice [93], Milciclib delivery can be enhanced into the brain in MDR1a/b and BCRP1 triple-knockout mice [94], suggesting that the anti-tumor efficacy of Milciclib in brain tumors can be enhanced by the regulation of ABC transporter activity. Evidently, there are many reports demonstrating that drug chemical delivery for brain tumors could be induced through the regulation of ABC transporter activity, thus suggesting the potential for the control of ABC transporter activity to control drug efficiency. This is a crucial point of consideration for the development of drug treatments for brain disorders.

## 3. Current Approaches to Increasing the Permeability of the BBB

**Figure 4:**
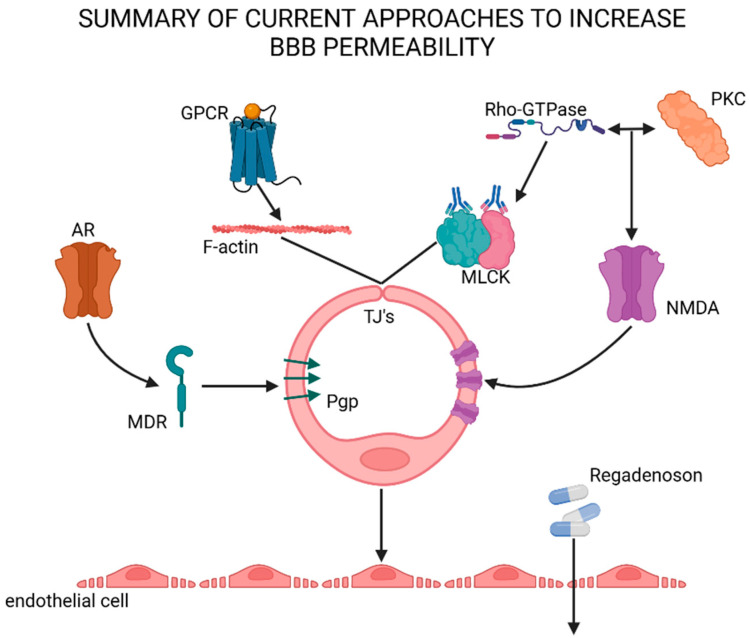
Schematic representation for approaches to increase BBB permeability, including the activation of AR and NMDA receptors, the increasing of junctional gaps and drug treatment such as Regadenoson. GPCR—G-protein-coupled receptor; TJs—tight junctions; NMDA—N-methyl-D-aspartate; PKC—protein kinase C; MLCK—myosin light chain kinase; Pgp—P-glycoprotein; AR—adenosine receptor; MDR—multi-drug resistance protein.

Recently, evidence has been provided that brain endothelial cells are activated by receptors that are expressed on the cell surface. This knowledge has been applied in attempts to increase BBB permeability and ultimately enhance the delivery of molecules from the peripheral system. Figure 4 demonstrates current approaches to increasing the permeability of the BBB, which will be discussed in detail in the literature to follow.

### 3.1. Activation of Receptors on the Brain Endothelial Cells

Most of the receptors that are expressed on the brain endothelial cells are G-protein-coupled receptors (GPCR). Activation of these receptors induces the influx of calcium into the brain endothelial cells, which can potentially increase the junctional gaps that lead to the increased permeability of molecules from the peripheral system [95]. Several studies have shown that GPCR activation can induce the reorganization of the F-actin, thereby following the restructuring of junctional molecules. For example, adenosine receptor activation has demonstrated the formation of polymerized actin in the brain endothelial cells [96]. Moreover, multiple studies have shown the disruptive effect of tight junctions in the brain endothelial cells, leading to the increased permeability of peripherally injected therapeutic drugs into the brain [97,98]. It was also shown that the increased permeability was mediated by the activation of Rho-GTPase and the subsequent activation of myosin-light chain kinase (MLCK) [99,100]. Moreover, the application of the FDA-approved drug Regadenoson (Lexiscan, Northbrook, IL: Astellas Pharma US, Inc) has shown promise for the activation of receptor signaling to increase the permeability of large molecules to the brain [101]. In contrast, the inhibition of this receptor and the upstream molecule that generates adenosine (CD73) could potentially block the entry of immune cells into the brain [102]. The activation of adenosine receptors (AR) causes the suppression of the expression and activity of multi-drug resistant (MDR) genes, including P-gp, resulting in the blocking of entry of anti-cancer drugs into the brain [103]. This demonstrates that the activation of AR can induce the suppression of P-gp expression by activating ubiquitination and migration to the insoluble fraction of this protein and subsequently induce increased accumulation of epirubicin, which is the substrate for P-glycoprotein [103]. These findings therefore additionally indicate that AR activation can induce paracellular and transcellular permeability enhancement. Brain endothelial cells also express receptors for neurotransmitters. Recent studies have shown that the activation of NMDA receptors on the brain endothelial cells can induce disruption of the cell’s barrier integrity in the brain, possibly suggesting increased permeability mediated by the activation of this receptor [104,105]. More recent studies have demonstrated that the activation of NMDA receptors in the brain endothelial cells could induce this increased permeability through the mediation of protein kinase C (PKC) activation and Rho-GTPase that downmodulates the function of lysosomal activity and potentially enhance the permeability of molecules that are readily transferred through RMT delivery [106]. It is yet to be elucidated which subtypes of NMDA receptors in the brain endothelial cells are affecting these changes, and how rapidly these responses are elicited.

### 3.2. Types of Carrier Systems to Increase Drug Delivery to the Brain

**Figure 5:**
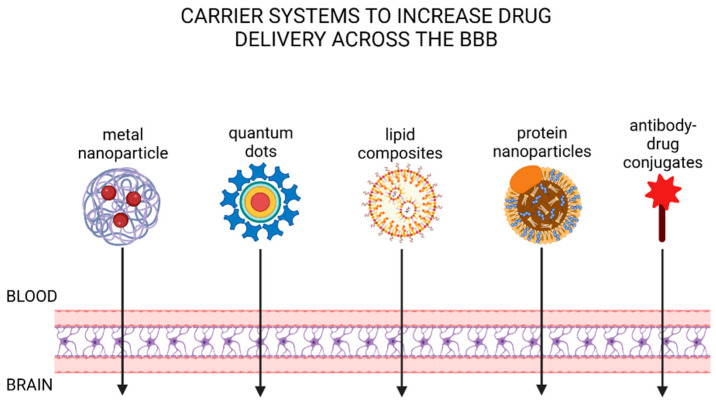
Schematic representation of carrier systems to increase drug delivery across the BBB. Methods described include metal nanoparticles, quantum dots, lipid composites, protein nanparticles and antibody-drug conjugates.

Brain endothelial cells selectively allow the delivery of molecules into the brain, which is mostly carried out through RMT or CMT [6]. Varieties of drug carriers including brain shuttles utilize these innate delivery systems of the brain endothelial cells to enhance the delivery of molecules to the brain by the application of bioengineering techniques incorporating the ligand or molecules with high affinity for the target receptors or carriers. There are multiple successful examples of these approaches to enhance molecular delivery including small molecules, therapeutics, and so on, including transferrin receptor (TfR) associated brain shuttles. In this section, we present various types of drug delivery systems and summarize recent advancements in methods aimed at enhancing the delivery of molecules through these pathways. Figure 5 summarizes the following systems in place to increase drug delivery across the BBB and into the brain as discussed below.

#### 3.2.1. Metal Nanoparticle (MNP)

MNPs possess physical (magnetic-active, optical-active) and chemical (functionalization, enzymatic reduction) properties, and are powerfully capable, despite their small size. MNPs are being investigated as a fascinating bio-medical platform, with their examined possibility for various clinical uses exceeding 50 years [107]. It is therefore no surprise that using MNPs for BBB penetration is a promising avenue for future studies, focusing on optimizing physicochemical properties for targeted therapeutic delivery to the brain. Unraveling the complexities and potential challenges, such as toxicity, associated with MNPs will assist in the accelerating focus on MNPs and the exhibition of variations in materials, sizes, and characteristics. MNPs can be made of gold, silver, zinc oxide, and iron oxide. Furthermore, when mixed with two different metal MNPs, or following the introduction of polymers into MNPs, they are called Janus particles and have highly dynamic potential in bio-medical applications [108]. They are speculated to be a ‘micro-swimmer’ with self-propelling motions and dual-target strategies by many types of fabrications. Among MNPs, gold nanoparticles (GNPs) have notably demonstrated less immune toxicity and safe biodistribution. Thus, GNPs are being thoroughly investigated for therapeutic diagnosis, anti-cancer properties, and antiviral uses in research and clinical fields [109,110].

GNPs can react with many functional groups (i.e., thiol groups) via ligand exchange and are decorated with cell-penetrating peptides, receptor-binding peptides, antibodies, and DNA [111,112]. Table 1 summarizes various studies on the utilization of MNPs and the corresponding strategies for BBB penetration in each study. In addition, as the diameter of the GNP can also be controlled from 1 nm to 100 nm based on chemical reactions, this provides promise that the synthesis of GNPs of a desired size to fulfill a specific purpose is possible. Therefore, various works in the literature have indicated size-dependent therapeutic effects, circulation time, bio-distribution and BBB penetration [113,114]. Research on developing safe and effective neurotherapeutic strategies is crucial. Overall, ongoing investigations aim to advance our understanding and applications of metal nanoparticles in addressing neurological disorders.

#### 3.2.2. Quantum Dots (QDs)

QDs are emerging chemo-physical nanoparticles in the bio-medical field and are also becoming increasingly popular in nano-semiconductors in the electrical engineering industry. The particles can emit self-fluorescence following UV excitation, while their emission wavelengths are varied depending on their sizes [126]. As they have desirable characteristics, QDs are being administrated in bio-imaging, clinical diagnosis, and photodynamic therapy (PDT) [127]. Regarding their photodynamic properties, QDs act as a photosensitizer, absorbing UV emittance and synthesizing reactive oxygen species (ROS). It subsequently causes the activation of receptors, thereby followed by caspase release, and finally the induction of cellular apoptosis [128,129].

**Table 2:** Recent research in utilizing QDs for BBB penetration in the treatment of brain diseases.

QDs	Target Diseases	Strategies for BBB Penetration	Animal	ECs	Biological Effects	Ref.
CQDs	AD	NIR photothermal effect	O (APP/PS1)	bEnd.3	CQD-based nanosystem mitigates Aβ neurotoxicity, enhances BBB permeability, and reduces Aβ deposition in AD.	[130]
CdSe/ZnS QDs	-	Mpr1 protein	-	hCMEC/D3	Mpr1-functionalized QD nanoparticles enhance BBB penetration, showing potential for drug delivery technology.	[131]
MoS2 QDs	AD	NIR photothermal effect	O (APP/PS1)	bEnd.3	MoS2 QDs exhibit targeted multi-effect therapy for AD, addressing ROS elimination and Aβ deposition.	[132]
CQDs	AD	-	O	-	SeCQDs offer multi-target therapy for AD by inhibiting Aβ aggregation and acting as a broad-spectrum antioxidant.	[133]
CQDs	GBM	LINTT1 peptide/Transferrin	O	-	ICG-derived CQDs enable red imaging of GBM cells, exhibit low toxicity, and demonstrate BBB penetration in zebrafish models.	[134]
GQDs	GBM	RVG peptide	O	bEnd.3	RVG-GQDs enhance brain tumor drug delivery, improve distribution, and achieve synergistic therapy potential.	[135]
SeQDs	AD	-	O	bEnd.3	SeQDs penetrate BBB, inhibit Aβ aggregation, reduce oxidative stress, and improve memory in AD mice.	[136]
GQDs	AD	Intranasal Delivery	O	-	CS/GQD NPs enhance memory recovery, target brain, and exhibit neuroprotective and anti-inflammatory effects in AD rats.	[137]
GQDs	AD	-	O (APP/PS1)	-	GQDs improve memory, reduce Aβ plaques, enhance neuron generation, and modulate inflammation in AD mice.	[138]
CQDs	AD	-	O	-	CQDs-MH inhibits tau aggregation, enhances delivery across the BBB, and holds therapeutic potential for AD treatment.	[139]

Abbreviations: CQDs—carbon quantum dots; AD—Alzheimer’s disease; MoS2 QDs—molybdenum disulfide quantum dots; GBM—glioblastoma; GQDs—graphene quantum dots; SeQDs—selenium quantum dots.

QDs are emerging chemo-physical nanoparticles in the bio-medical field and are also becoming increasingly popular in nano-semiconductors in the electrical engineering industry. The particles can emit self-fluorescence following UV excitation, while their emission wavelengths are varied depending on their sizes [126]. As they have desirable characteristics, QDs are being administrated in bio-imaging, clinical diagnosis, and photodynamic therapy (PDT) [127]. Regarding their photodynamic properties, QDs act as a photosensitizer, absorbing UV emittance and synthesizing reactive oxygen species (ROS). It subsequently causes the activation of receptors followed by caspase release and finally the induction of cellular apoptosis [128,129]. Despite the gravity of this novel process, ROS could be a major problem in terms of toxic side-effects to mammalian cells. QDs must therefore be validated with clinical data including distributions, circulation time, and excretion [140]. As is the case with MNPs, various conjugations between QDs with biomolecules and ligands are also observed [141]. Additionally, transferrin is adopted on the surface of QDs; hence, through in vitro models, their ability for transport and cellular uptake into endothelial cells has been demonstrated [142].

Table 2 presents an assortment of examples of QDs along with detailed descriptions of the respective studies. The future of research into QDs for BBB penetration is promising, focusing on optimizing their unique properties for targeted drug delivery to the brain. Essential considerations include investigating potential challenges like toxicity and long-term effects to ensure the development of safe and effective therapeutic applications. Overall, QDs hold the potential to revolutionize drug delivery and diagnostics in neurotherapeutics.

#### 3.2.3. Lipid Composites

Lipid composites are considered highly potent carriers due to the use of several natural and synthesized phospholipids postulates. For example, the COVID-19 RNA vaccines were also manufactured based on lipid composites, bio-safe capsulation of RNAs and stealth properties [143]. Extracellular vesicles (EVs) are cell-derived lipid composites with a lipid bilayer as well as exosomes, micro-vesicles, exosomes and apoptotic bodies. EVs play an integral role in cell communication and signal conduction in all cell types [144]. Exosomes have been focused on as the third generation of drug delivery platforms since they are cell-derived and can communicate with other cells with ligands on their surfaces. Notably, they are internalized into cells, and excrete signaling molecules as part of a conventional drug delivery system [145]. For example, exosomes were dissected within a targeting glioma tumor, successfully engineering ligands on exosomes [146,147].

Liposomes are common artificial lipid composites, which are composed of various functional phospholipids. As liposomes are easily synthesized, they have been researched as platforms of drug delivery systems for many years [148]. Liposomes are also manipulated with tunable phospholipids, expanding the collaboration with biomolecules. Specifically, transferrin-embedding liposomes were evaluated with regard to BBB penetration and subsequent therapeutic effects [149].

Solid lipid nanoparticles (SLNs) also form a part of the usual lipid composites such as liposomes; however, they are named differently as they have a lipid crystal lattice core in their lipid monolayers. Oligonucleotides, which are negatively charged, are usually contained in SLNs due to their interactions with cationic lipids. The first RNA-SLN drug, Patisiran, was approved by the FDA in 2018 [150]. Additionally, both consisting of amphiphilic characteristics, hydrophobic and hydrophilic drugs can be capsulated. This may indicate that lipid composites, especially SLNs, are candidates for universal drug carriers [151]. In a study [152] using transferrin (Tf) as a brain-targeting ligand, both in vitro and in vivo protein coronas had distinct effects on receptor targeting, lysosomal escape, and BBB transcytosis. The study suggests a potential role of apolipoproteins, particularly apolipoprotein A-I, in aiding NPs to traverse the BBB, providing valuable insights for brain-targeted delivery development. Collectively, our results confirm the pivotal involvement of the protein corona in the BBB transcytosis of Tf-NPs, opening up new avenues for the future development of brain targeting strategies. Furthermore, ongoing investigations involve the modification of numerous BBB-permeating peptides on SLN surfaces using diverse linkers. This approach broadens the spectrum of research within this domain. The diverse range of linkers utilized adds a layer of complexity, reflecting the nuanced strategies employed to enhance BBB penetration. This collective effort signifies a significant expansion in the depth and breadth of research aimed at advancing our understanding of brain-targeted drug delivery.

#### 3.2.4. Protein Nanoparticles

Many researchers have tried to develop new therapeutic interventions for brain injuries such as small chemotherapeutic molecules and RNAs. While many of these interventions have shown promise, being able to bypass the BBB to target specific brain regions where disease or injury has occurred is the most arduous aspect for any researcher. Artificial composites are highly unstable in physiological conditions (such as in vivo) and may potentially cause severe immune responses in patients as well as interfere with alternative therapeutic modalities such as chemotherapy [153]. For these reasons, human- or animal-derived protein-based nanoparticles can be an effective way to reduce tumor activity [154,155]. Serum albumin makes up approximately 55% of plasma in the blood, has been shown to interact with transcellular proteins, such as secreted protein acidic, and is rich in cysteine (SPARC) and gp60 in endothelial cells around tumor vessels [156,157]. It implies that albumin nanoparticles can stay for a long time without interacting and interfering with other blood cells.

**Table 3:** Recent research in utilizing protein nanoparticles for BBB penetration in the treatment of brain diseases.

Protein Nanoparticles	Target Diseases	Strategies for BBB Penetration	Animal	ECs	Biological Effects	Ref.
Albumin NPs	Glioma	LMWP peptide	O	bEnd.3	Albumin nanoparticles enable enhanced brain-targeted drug delivery, exhibiting synergistic therapeutic effects in glioma.	[158]
GS NPs	-	SynB peptide	O	BCECs	SynB-PEG-GS nanoparticles enhance BBB penetration, exhibiting superior brain delivery compared to PEG-GS.	[159]
M-CA NPs	Glioma	menthol	O	BCECs	Menthol-modified casein nanoparticles enhance glioma-targeted drug delivery, improving therapeutic efficacy safely.	[155]
Ferritin NPs	Glioma	HFn and HFn receptor binding	O	bEnd.3	Ferritin NPs cross BBB, selectively target glioma cells, and induce lysosomal-mediated glioma cell death.	[160]
Lipoprotein NPs	AD	ApoE and LDLR binding	O (SAMP8)	-	ApoE3–rHDL nano medicine accelerates Aβ clearance, reduces deposition, and mitigates AD-associated pathology.	[161]

Abbreviations: GS NPs—gelatin-siloxane nanoparticles; BCECs—brain capillary endothelial cells; M-CA NPs—menthol-modified casein nanoparticles.

Lipoproteins, nanoparticles that naturally exist in the human body, possess various advantages due to their small size. Furthermore, through fusion with apolipoprotein, lipoproteins can bypass the BBB and be delivered to the brain. High-density lipoprotein (HDL) is known to bind to amyloid-β protein, its aggregation a known hallmark of dementia, and can thereby be used as a drug delivery system [161]. Human H-ferritin (HFn) has the capability to cross the BBB by binding to transferrin receptor 1 (TfR1), which is plentiful in endothelial cells and commonly upregulated in tumors. This interaction facilitates the entry of HFn into cells through the process of endocytosis. The tumor-targeting capacity of HFn-based nanoparticles was found to double after traversing the blood–brain barrier (BBB) in both an in vitro transcytosis assay and an in vivo orthotopic glioma model. Additionally, a reduction in tumor growth was observed in the mouse model [162]. In addition, various studies on protein nanoparticles of diverse types have been conducted, and examples along with the mechanisms for BBB penetration are systematically arranged in Table 3. Future research on protein nanoparticles for BBB penetration focuses on optimizing size and surface characteristics to enhance targeted drug delivery to the brain. Addressing challenges like immunogenicity and biodistribution is crucial for ensuring safe and effective therapeutic applications. These advancements not only offer promise for innovative drug delivery in neurotherapeutics but also hold the potential for revolutionizing the treatment of neurological disorders.

#### 3.2.5. Antibodies as Drugs

Antibody–peptide conjugates (APCs) are similar to antibody–drug conjugates (ADCs) but are different in concept. Peptides are used as tools to target cancer cells to cross the BBB; however, drugs implicated in ADCs are used as cargo to kill tumors. ADCs, comprising recombinant monoclonal antibodies covalently linked to cytotoxic chemicals via synthetic linkers, are effective treatments. However, brain treatment is limited due to the blood–brain barrier obstruction. In addition, the breakdown of the BBB that is initiated by neurodegenerative diseases such as amyloid deposition in AD is less heterogeneous. Such immunoconjugates combine the antitumor potency of highly cytotoxic small-molecule drugs (300–1000 Da) with the high selectivity, stability and favorable pharmacokinetic profile of monoclonal antibodies. As mentioned previously, among the many methods for crossing the BBB, there are studies using peptides that can be easily linked to antibodies. A case exists in which viral COVID-derived peptides were linked to antibodies to increase BBB penetration [163].

RMT entails several steps that are initiated by ligand–receptor binding and endocytosis and subsequently guided by intracellular trafficking and vesicle fusion to arrive at a targeted location. RMT through the LRP family has been applied in treating brain metastatic tumors. The receptor, particularly low-density-lipoprotein-receptor-related protein 1, is highly expressed on the surface of the BBB and pre-clinical research has shown angiopep-2 as a key facilitator in this transcytosis process. Angiopep-2, widely known as a BBB-crossing peptide, is conjugated to an antibody, and it was found that antibodies with angiopep-2 accumulate in the brain in mice [164]. One of the most well-known examples of RMT-mediated antibody therapeutics is the TfR ligand-based brain shuttle. Various works in the literature investigating TfR ligand-based antibody therapeutic development and application and its effectiveness in enhancing drug delivery have been introduced. Currently, it is also being used as one of the tools for studying the role of the endosomal trafficking pathways in determining the penetration level of antibody therapeutics into the brain. According to the recent study by Villasenor et al., it was suggested that the mode of action of this brain shuttle is mediated by the regulation of endosomal sorting tubule that can selectively allow the entry of this therapeutic shuttle, which has not been previously explored [34].

One of the most recent applications of this brain shuttle is TREM2-specific antibody therapeutics, which are incorporated into this brain shuttle. In Ads, TREM2, a receptor found on microglia, plays a pivotal role in AD by regulating immune responses and facilitating the clearance of amyloid-beta plaques. Mutations in the TREM2 gene are linked to an elevated risk of Alzheimer’s, highlighting its significance in neuroinflammation modulation. Exploring TREM2’s impact may offer promising avenues for developing novel therapies for AD. The study [165] introduces ATV:TREM2, a high-affinity antibody designed to activate human TREM2, with a monovalent TfR binding site for blood–brain barrier transcytosis. Administered in mice peripherally, ATV:TREM2 shows enhanced brain biodistribution and signaling compared to a standard anti-TREM2 antibody. In iPSC-derived microglia and an Alzheimer’s mouse model, ATV:TREM2 has the potential to induce microglial proliferation, improve mitochondrial metabolism, and boost brain microglial activity, presenting a promising strategy for addressing Alzheimer’s-related brain hypometabolism.

One of the recently developed brain shuttles is the insulin-like growth factor 1 receptor (IGF1R) binding motif conjugated brain shuttle [166]. IGF1R has an essential role in the formation of the synapses in the brain that is mediated by its receptor tyrosine kinase-dependent signaling pathway. This receptor is highly expressed in the brain endothelial cells. A recent study has shown an antibody-based shuttle that was engineered to express the IGF1R binding motif. In a study that was performed in vitro and in vivo, it was demonstrated that this brain shuttle can cross the BBB effectively and its delivery can be increased in the animal models of Parkinson’s Disease (PD) showing its promise as an alternative antibody-based brain shuttle [166]. However, more detailed mechanisms of its increase in deliverability would be required to broaden its application [166].

The majority of existing studies have not adequately elucidated the process of BBB penetration, which requires further in-depth study to be utilized for the development of a better antibody-based brain shuttle. Effective utilization of APCs holds promise for enhancing BBB permeability, providing a potential avenue for advancing our understanding and therapeutic strategies for AD. Future studies on antibody drugs and blood–brain barrier (BBB) penetration aim to enhance their ability for targeted drug delivery to the brain. Overcoming challenges in antibody design, specificity, and potential side effects is essential for developing safe and effective therapeutic applications. These endeavors mark the forefront of advancing neurotherapeutics and addressing diverse neurological disorders.

#### 3.2.6. Serum Albumin and RBC Coated Nanoparticles

In drug delivery systems, the use of externally artificial substances not naturally present in the body has been common, leading to typical challenges of elimination by various immune cells in the bloodstream. This has often resulted in significant inflammatory responses and adverse effects. To overcome this challenge, the utilization of bovine or human serum albumin (SA) and red blood cell (RBC) coated particles in drug delivery enables evasion of immune cells within the bloodstream. Consequently, this approach diminishes the probability of immune recognition and clearance, thereby optimizing drug delivery efficiency. Many studies have used biologically derived proteins and when avoiding the immune system, have reduced the inflammation from capsules of a drug [167]. In this case, the most promising protein is SA, as designed nanoparticles with albumin have interestingly yielded promising results. Using a BSA coating on the surface of drug-loaded nanoparticles has been confirmed to increase survival in the blood [168,169]. In addition, studies have shown an attempt to mimic the red blood cell membrane and use it as a drug delivery system. This technique allows for the drug carrier to evade the immune response and stay in the blood for a prolonged duration [170]. Table 4 summarizes studies utilizing nanoparticles encapsulated by albumin and RBC membranes.

Those drug delivery systems showed various efficiencies of their permeable effect through the BBB. The summary of increased BBB penetration efficiency in various systems is presented in Table 5.

## 4. Novel Strategies of Drug Carrier Design

**Figure 6:**
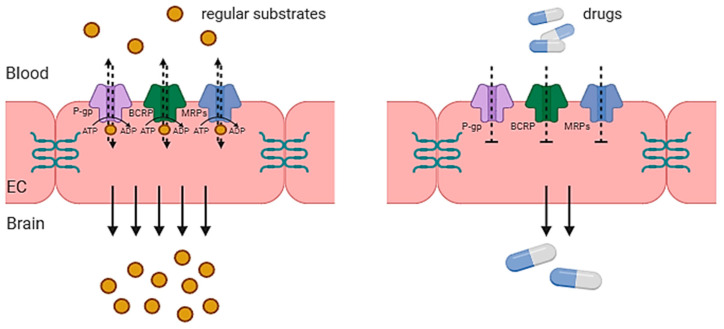
Schematic illustration of the impediment of drug delivery to the brain, due to ABC transporter activity. EC—endothelial cell; ATP—adenosine triphosphate; ADP—adenosine diphosphate; P-gp—P-glycoprotein; BCRP—breast cancer resistance protein; MRPs—multi-drug resistance protein. Adapted from Gil-Martins et al. [177].

In the domain of drug carrier design, recent endeavors have introduced innovative strategies to enhance drug delivery effectiveness. These approaches encompass a multifaceted exploration including the extension of circulation time, considerations of size and shape dependencies, utilization for diagnostic and imaging purposes, investigations employing molecular dynamics simulations to understand blood–brain barrier (BBB) crossing, and critical assessments of existing methodologies’ constraints. Such comprehensive efforts underline the dynamic landscape of drug carrier design, integrating diverse disciplines to tackle challenges and optimize therapeutic outcomes. Figure 6 illustrates ABC transporters and the impediment of drug delivery to the brain.

In addition, Table 6 summarizes the current ABC transporters with the known substrates and inhibitors that are expressed on the BBB and are under study for modification.

### 4.1. Increased Circulation Time

The clearance of nanoparticles is primarily influenced by the patient’s immune system. Moreover, macrophages are recruited to handle foreign bodies in the reticuloendothelial system, inadvertently leading to the opsonization of nanomedicine. One significant parameter recognized by macrophages is charge interaction [178]; hence, managing the surface charge of nanoparticles to be around zero is imperative [179,180,181]. This is because a zeta potential close to zero prevents nonspecific binding, allowing structures to remain intact for extended periods. However, when the zeta potential of nanoparticles in drug delivery systems becomes positive, challenges regarding circulation time may arise [182,183]. Nanoparticles with positive zeta potentials tend to interact more with negatively charged components in the bloodstream, such as erythrocytes and plasma proteins, accelerating their clearance [184]. Additionally, aggregated nanoparticles with positive zeta potentials may block blood vessels, leading to the formation of emboli and causing adverse effects such as ischemia or thrombosis [185]. Nonetheless, positive zeta potentials may also confer advantages in drug delivery to the brain, as nanoparticles with a positive surface charge can cross the blood–brain barrier through adsorptive-mediated transcytosis (AMT) [186]. Thus, assessing the zeta potential of nanoparticles is crucial for understanding their interaction with biological systems and optimizing their applications in drug delivery.

The absorption of nanoparticles varies across different tissues and organs, with size playing a crucial role in this process. Larger nanoparticles tend to be efficiently absorbed by the liver, owing to their specialized filtration mechanisms and fenestrated endothelium [113]. In contrast, smaller nanoparticles exhibit prolonged circulation times in the bloodstream, enhancing their potential for systemic delivery to target tissues, especially the brain [187]. The renal clearance of nanoparticles is influenced by their size, with smaller particles being more readily excreted via the kidneys [188]. The lymphatic system plays a crucial role in the absorption of nanoparticles, particularly those of larger sizes, which are more likely to be transported via lymphatic vessels to systemic circulation. The blood–brain barrier presents a challenge for nanoparticle delivery, with smaller particles showing potential for crossing this barrier and accessing the central nervous system [189]. Nanoparticle size significantly influences their uptake by tumor tissues, with smaller particles often demonstrating enhanced penetration and accumulation due to the enhanced permeability and retention (EPR) effect [190,191,192]. Larger nanoparticles may face challenges in penetrating the dense extracellular matrix of tumors, limiting their uptake and therapeutic efficacy [193]. Tailoring nanoparticle size to exploit tumor-specific characteristics can improve their uptake and retention within tumor tissues, ultimately enhancing the effectiveness of cancer therapeutics. Understanding the size-dependent absorption kinetics of nanoparticles in various tissues and organs is essential for designing targeted and efficient nanoparticle-based drug delivery systems.

The lipidization of drugs to enhance brain uptake is very nonspecific; therefore, a carrier system developed for organ selectivity and increased circulation time is of great interest. Such a delivery system, using transferrin, for example, would take advantage of the high-density receptors as well as the ability of these receptors to shuttle molecules across the BBB. Research has demonstrated that in rats, the anti-transferrin receptor antibody OX-26 and antibody-methotrexate (MTX) conjugates bind to capillary endothelial cells in a dose and time-dependent manner [194].

### 4.2. Size and Shape Dependence

As previously mentioned, the shape and size of nanoparticles have been shown to significantly affect their biodistribution and pharmacokinetic profile [195]. RNA nanotechnology, in which ligands and scaffolds can be composed solely of RNA, has become increasingly popular due to promising advancements in overcoming the current challenges in nanotechnology. RNA nanoparticles can be engineered to a unique size and shape while retaining the high thermostability required for in vivo applications [196]. This is of importance in the optimization of drug delivery, where nanoparticles have to be consistent in assembly to ensure the pharmacokinetics and biodistribution in vivo. The study conducted by Jasinski et al. showed that there is a strong association between increased size and increased circulation time using RNA nanosquares that were 5, 10 and 20 nm along each edge. In addition, increasing the nanoparticle size increases its hydrodynamic viscosity; therefore, increased interactions of larger squares with the surrounding environment, water, for example, will slow down its diffusion and contribute to the increase in circulation time [196].

The targeting of nanoparticles to the endothelium specifically is limited by several factors. The target size, namely the area of diseased endothelium, is often much smaller than healthy endothelium. Moreover, the ability of particles to avoid immune clearance and accumulate at a region of interest depends on parameters such as size, shape, surface chemistry and flexibility. In vivo biodistribution research has demonstrated the benefits of elongated particles in targeting the endothelium and endothelial cells. For example, greater specific attachment exhibited by rod-shaped particles offers several advantages in the field of drug delivery [196,197]. Specifically, it has become evident that nanoparticle shape significantly influences biodistribution, intravascular and transvascular transport, and ultimately, their efficacy in targeting elusive cancer sites. This underscores the importance of shaping nanoparticles to optimize their potential as cancer-targeting agents [198].

### 4.3. Diagnosis and Imaging

There are different ways to bypass the BBB. The presence of specific transport systems within the capillary endothelial cells, such as those for amino acids, transferrin, glucose and insulin ensures that the brain receives all compounds required for maintenance [194]. In chemotherapeutic examples, iatrogenic agents or intrathecal drug administration are also capable of crossing the BBB. Other methods used to penetrate the BBB may involve intracerebral implantation, convection-enhanced distribution, Mannitol and the endogenous transport systems, namely CMT and RMT. However, mechanisms for BBB delivery are not sufficient for targeting and treatment of malignant gliomas, which are the most aggressive and lethal primary brain tumors in adults. Nanotechnology-based delivery systems are being studied for the effective treatment of various brain tumors and the reduction of side effects, therefore enabling the combination of targeting, drug loading and drug release in a large capacity. A nanomaterial that can potentially be used for both the treatment and imaging of a disease would be of great interest. For example, magnetically responsive magnetite (Fe_3_O_4_) and maghemite (Fe_2_O_3_)-based crystalline particles can be readily prepared as nanoscale-sized formulatio1ns (3.0–100.0 nm).

Techniques to deliver drugs across the BBB include magnetic methods such as the use of iron oxide nanoparticles (IONPs). Moreover, a study described a method that applied a radio frequency field to cause the heating of commercial IONPs which had been administered via the middle cerebral artery using a catheter. The technique enabled large dye molecules which were injected prior to IONPs to cross the BBB. As mentioned before, common receptors for BBB transcytosis are the TfR, which is expressed by brain capillary ECs and serves as RMT of iron-bound transferrin through the BBB. In another study, magnetite-coupled dextran-spermine coated NPs were conjugated to transferrin and facilitated the crossing of the BBB. Common magnetic imaging methods include magnetic resonance imaging (MRI), magnetic particle imaging (MPI) as well as multimodal imaging which combines these two techniques [199].

### 4.4. Molecular Dynamics Simulation of BBB Crossing

Utilizing molecular dynamics (MD) simulation for BBB crossing is essential for comprehending the nuanced processes involved. Through MD simulation, researchers can dissect the intricate molecular interactions between nanoparticles and BBB components, offering detailed insights into their dynamic behavior [200,201]. This method facilitates the prediction and refinement of nanoparticle properties, thus optimizing their ability to penetrate the BBB and informing the development of more efficacious drug delivery systems tailored for neurological disorders.

Studies have demonstrated the use of molecular dynamics (MD) for crossing magnetic nanoparticles through the BBB. A prominent example includes putting insulin receptors on the BBB for the transport of nanocapsules. The first simulation conducted to elucidate the crossing of gold nanoparticles through the BBB was carried out for an uncoated 2 nm diameter nanoparticle crossing a 100A × 100A membrane using the SMD method. The study indicated that an alternative magnetic field is required to make an equivalent force for the process to be successful [202]. Following initial research, magnetic nanoparticles have been utilized for crossing through a simplified BBB model for glioblastoma multiforme treatment. A study conducted by Gkountas et al. demonstrated a 45% increase in BBB permeability for magnetic nanoparticles (MNPs) of up to 100 nm with an applied magnetic field. This was achieved using 3D Navier–Stokes equations that are solved in the margin of a blood vessel along with a discrete model of MNPs with various acting forces. These results are then compared with experimental measurements that could ultimately predict the flow behavior [203]. In addition, studies have developed a physiologically based pharmacokinetic (PBPK) model for intraperitoneal (IP) injected superparamagnetic iron oxide nanoparticles coated by gold and conjugated with poly (ethylene glycol) (PEG) (SPIO-Au-PEG NPs) in mice. The model was able to predict the in vivo biodistribution of SPIO-Au-PEG NPs under the exposure of an external static magnetic field and demonstrated that modifications with insulin showed an improvement in brain bioavailability by 24.47% in comparison to control groups that were not insulin-treated. This provides promise for future in vivo studies for non-invasive targeted drug delivery to the brain [204].

### 4.5. Limitations of Current Methods

While current methods show great promise for the future of drug delivery into the brain, there are still various limitations that may impede techniques’ capabilities of being deemed completely successful. For example, for magnetic field research, high area ratios of the BBB show an unchanged permeability when the magnetic field is applied, indicating that magnetic force alone cannot drive the MNPs towards the BBB, but that they are driven by blood flow instead. In the same light, the small size of the magnetic core is a limitation for the magnetic targeting efficiency. With regard to RMT, this process might face challenges related to designing appropriate drug carriers that can effectively target specific receptors and navigate a complex cellular environment. Approved drugs such as aducanumab and lecanemab, as well as drugs that are ongoing trials, aim to prevent the buildup of neurodegenerative proteotoxicty and eventually slow disease progression [205]. Moreover, there is the long-standing issue that most optimization for brain research is limited to non-invasive resources outside of the living body. It is therefore crucial that strategies to enhance molecular drug delivery to the brain be further explored.

## Figures and Tables

**Table 1:** Recent research in utilizing MNPs for BBB penetration in the treatment of brain diseases.

MNPs	Target Diseases	Strategies for BBB Penetration	Animal	ECs	Biological Effects	Ref.
AuNPs	AD	-	-	bEnd.3	Reduced amyloid aggregation, minimized cellular damage, Alzheimer’s therapeutic potential.	[115]
AuNPs	-	FUS	O	bEnd.3	Nanoparticle delivery through widened BBB. Size-dependent permeation, optimal size for brain delivery determined.	[116]
IONPs	GBM	-	-	bEnd.3	Enhanced GBM treatment using magnetic nanoparticles. Improved drug permeability, targeted cytotoxicity, apoptosis induction.	[117]
AuPdNPs	AD	P80	-	bEnd.3	Quercetin-modified nanoparticles induce autophagy, accelerate Aβ clearance, and protect against cytotoxicity in AD.	[118]
IONPs	AD	-	O (APP/PS1)	bEnd.3	Sialic acid-coated NPs detect Aβ plaques noninvasively. Overcome BBB, high selectivity, promising for in vivo detection.	[119]
IONPs	-	magnetic field	-	bEnd.3	AMF-induced hyperthermia enhances nanoparticle BBB association and flux, involving temperature-related mechanisms.	[120]
IONPs	Glioma	-	O	-	Multifunctional NPs enhance drug delivery, BBB penetration, and therapeutic efficacy against glioma.	[121]
AuNPs	Glioma	TAT peptide	O	-	TAT-Au NPs cross BBB, deliver anticancer drugs, enhance glioma therapy, and improve brain tumor imaging.	[122]
AuNRs	AD	angiopep-2 peptide	-	hCMEC/D3	BBB-oC with neurovascular network facilitates GNR-PEG-Ang2/D1 entry, showing potential for enhanced drug delivery.	[123]
AuNPs/AuNRs	ND	Transferrin peptide, NIR irradiation	O	CD34+ cells	AuNRs efficiently cross BBB and accumulate in neurogenic niches, promoting targeted neurogenesis.	[124]
AuNPs	AD	-	O (APP/PS1)	-	Chiral gold nanoparticles inhibit Aβ aggregation, cross BBB, and show therapeutic potential for AD.	[125]

Abbreviations: BBB—blood–brain barrier; ECs—endothelial cells; AuNPs—gold nanoparticles; AD—Alzheimer’s disease; IONPs—iron oxide nanoparticles; GBM—glioblastoma; PdNPs—palladium nanoparticles; AuNRs—gold nanorods.

**Table 4:** Recent research in utilizing albumin- and RBC-coated nanoparticles for BBB penetration in the treatment of brain diseases.

Coating Type	Strategies for BBB Penetration	Biological Effects	Ref.
Albumin	SPARC and gp60 binding	Novel albumin-coated NPs enhance GBM therapy with improved BBB permeation and reduced hemolytic toxicity.	[171]
		Albumin-coated NPs deliver the TRAIL gene, inducing glioma apoptosis and inhibiting tumor growth.	[172]
		Cationic BSA-conjugated NPs efficiently deliver neuroprotective effects in ischemic stroke by modulating inflammatory pathways.	[173]
RBC membrane	-	RBC-membrane-coated NPs reprogram the GBM microenvironment for enhanced immunotherapy.	[174]
	CDX peptide	BBB-targeted drug delivery system utilizes RBC-membrane-coated NPs for enhanced therapeutic efficacy.	[175]
	RGD peptide	A biomimetic nanodevice co-encapsulates chemotherapeutic drugs, demonstrating superior tumor growth inhibition with reduced side effects.	[176]

Abbreviations: GBM—glioblastoma; TRAIL—tumor necrosis factor-related apoptosis-inducing ligand.

**Table 5:** Quantitative analysis of BBB penetration enhancement based on carrier types.

Types of Carrier Systems	Strategies for BBB Penetration	ECs/ Animals	Fold Increases	Ref.
MNPs	FUS	bEnd.3	9.5	[158]
	P80	bEnd.3	2.5	[159]
	TAT peptide	Mouse	4.8	[155]
QDs	NIR photothermal effect	Mouse	1.6	[160]
Protein NPs	LMWP peptide	bEnd.3	2.5	[161]
	SynB peptide	BCECs	2.0	[161]

Abbreviations: BBB—blood–brain barrier; ECs—endothelial cells; fold increases—BBB penetration efficiency increases after decoration of NPs; BCECs—brain capillary endothelial cells.

**Table 6:** Transporters that are expressed on the BBB with their substrates and inhibitors.

ABC Transporter	Substrates		Inhibitors
P-glycoprotein	Anticancer drugs	Doxorubicin, Daunorubicin, Vinblastine, Vincristine, Etoposide, Teniposide, Paclitaxel, Methotrexate	Verapamil, Cyclosporin A, Quinidine, Quinine, Amiodarone PSC-833, Elacridar, VX-710, Dexverapamil ONT-093, Zosuquidar, Tariquidar, Laniquidar
	Immunosuppressive agents	Cyclosporine A	
	Analgesics	Morphine	
	Cytokines	IL-2, IL-4, IFN-y	
	Antiepileptic drugs	Phenytoin, Carbamazepine, Lamotrigine, Phenobarbital, Felbamate, Gabapentin, Topiramate	
	Antibiotics	Erythromycin, Valinomycin, Tetracyclines, Fluoroquinolones	
	Antidepressants	Amitryptiline, Nortryptiline, Doxepin, Venlafaxine, Paroxetine	
	Calcium channel blocker	Verapamil	
MRP1	Anticancer drugs	Etoposide, Teniposide, Doxorubicin, Leukotriene C4, D4, E4, Daunorubicin, Methotrexate Glutathione, Glucuronide, sulfate conjugates	Sulfinpyrazone, Probenecid, MK571, LTC4, some P-gp inhibitors
MRP2	Same as above		
MRP3	Organic anion transporter with considerable overlap in substrates of MRP1 and MRP2		Sulfinpyrazone, Probenecid, Indomethacin
MRP4	Anticancer drugs	Methotrexate, 6-mercaptopurine, thioguanine	
MRP5	cGMP, cAMP, 6-mercaptopurine, Thioguanine, Fluorescein		Probenecid, Trequensin, Sildenafil
	BQ-123		
BCRP	Anticancer drugs	Overlap with P-gp, MRP1 and MRP2 Anthracyclines, Mitoxantrone, Bisantrene, Camptpthecins topotecan, SN-38, Prazosin	GF120918, Fumitremorgin C (FTC)

Abbreviations: IL—interleukin; IFN-y—interferon gamma; LTC4—leukotriene 4; MRP—multidrug resistance protein; BCRP—breast cancer resistance protein; cGMP—cyclic guanosine monophosphate; cAMP—cyclic adenosine monophosphate.

## Data Availability

Data are contained within the article.
